# Melatonin protects rat cerebellar granule cells against electromagnetic field-induced increases in Na^+^ currents through intracellular Ca^2+^ release

**DOI:** 10.1111/jcmm.12250

**Published:** 2014-02-18

**Authors:** Dong-Dong Liu, Zhen Ren, Guang Yang, Qian-Ru Zhao, Yan-Ai Mei

**Affiliations:** School of Life Sciences, Institutes of Brain Science and State Key Laboratory of Medical Neurobiology, Fudan UniversityShanghai, China

**Keywords:** melatonin, ELF-EMF, Na^+^ currents, Ca^2+^ release, cerebellar granule cells

## Abstract

Although melatonin (MT) has been reported to protect cells against oxidative damage induced by electromagnetic radiation, few reports have addressed whether there are other protective mechanisms. Here, we investigated the effects of MT on extremely low-frequency electromagnetic field (ELF-EMF)-induced Na_v_ activity in rat cerebellar granule cells (GCs). Exposing cerebellar GCs to ELF-EMF for 60 min. significantly increased the Na_v_ current (*I*_Na_) densities by 62.5%. MT (5 μM) inhibited the ELF-EMF-induced *I*_Na_ increase. This inhibitory effect of MT is mimicked by an MT_2_ receptor agonist and was eliminated by an MT_2_ receptor antagonist. The Na_v_ channel steady-state activation curve was significantly shifted towards hyperpolarization by ELF-EMF stimulation but remained unchanged by MT in cerebellar GC that were either exposed or not exposed to ELF-EMF. ELF-EMF exposure significantly increased the intracellular levels of phosphorylated PKA in cerebellar GCs, and both MT and IIK-7 did not reduce the ELF-EMF-induced increase in phosphorylated PKA. The inhibitory effects of MT on ELF-EMF-induced Na_v_ activity was greatly reduced by the calmodulin inhibitor KN93. Calcium imaging showed that MT did not increase the basal intracellular Ca^2+^ level, but it significantly elevated the intracellular Ca^2+^ level evoked by the high K^+^ stimulation in cerebellar GC that were either exposed or not exposed to ELF-EMF. In the presence of ruthenium red, a ryanodine-sensitive receptor blocker, the MT-induced increase in intracellular calcium levels was reduced. Our data show for the first time that MT protects against neuronal *I*_Na_ that result from ELF-EMF exposure through Ca^2+^ influx-induced Ca^2+^ release.

## Introduction

Several studies have noted that exposure to extremely low-frequency electromagnetic fields (ELF-EMF) alters animal behaviours and causes biological effects, including changes in gene expression, the regulation of cell survival and the promotion of cell differentiation [1–3]. In addition, exposure to EMF induces changes in cerebral blood flow in old Alzheimer's mice [4]. Enzyme activity in cytosol or at the membrane and subsequent alterations in intracellular signalling are found in lymphoma B cells and Chinese hamster lung cells upon exposure to ELF-EMF [5,6]. Extremely low-frequency electromagnetic fields can also modify the biophysical properties of cell membranes as shown by changes in the membrane permeability of carbonic anhydrase [7] and stimulation of the activity of Ca^2+^-activated potassium channels *via* increases in Ca^2+^ concentration and voltage-gated calcium channels [3,8,9]. We recently reported that ELF-EMF exposure significantly activated the voltage-gated sodium (Na_v_) channels of cerebellar GCs [10]. This activation is mediated by an increase in the intracellular concentration of arachidonic acid and involves EP receptor–mediated activation of the cAMP/PKA signalling pathway [10].

Melatonin (MT), which is synthesized and primarily secreted by the pineal gland, participates in many important physiological functions, including the control of seasonal reproduction, and influences the immune system and circadian rhythms [11,12]. *In vitro* and *in vivo* studies have revealed that MT and its metabolites can reduce oxidative stress-induced damage to proteins, lipids and nucleic acids in the presence of free radicals because of its free radical-scavenging properties [13–15]. Because it has been postulated that EMF exposure can affect the function of biological systems by inducing oxidative damage, the effects of MT on EMF-induced oxidative damage, cancer risk and neurodegeneration have been investigated [16,17]. Besides its direct free radical-scavenging properties, MT has been shown to modulate apoptosis caused by wireless (2.45 GHz)-induced oxidative stress through cation channels, such as transient receptor potential (TRP) and voltage-gated Ca^2+^ channels in neurons and transfected cells [18,19]. In addition, MT modulates the delay in outward rectifying K^+^ channels resulting in the promotion of cerebellar GC migration or the protection of cerebellar GCs against apoptosis [20,21]. However, there have been relatively few studies concerning the effect of MT on Na^+^ channels, especially EMF-induced Na^+^ channels activity.

Voltage-gated sodium channels are one of the primary classes of ion channels responsible for driving neuronal excitability in both the central and the peripheral nervous system. Voltage-gated sodium channels are clinically important because they play an important role in the generation of neuronal activity, and alterations in Na_v_ channels are key factors in a number of pathologies [22]. Previous studies from other groups have revealed that Na_v_ channels participate in the rising phase of the neuronal action potential and contribute to many cellular functions, including apoptosis, motility and secretory membrane activity [22,23]. Moreover, EMF exposure was recently reported to modulate neuronal excitation and neurogenesis, which may be related to Na_v_ channel activity [24,25]. Our previous data have demonstrated that ELF-EMF exposure significantly activates the Na_v_ channels of cerebellar GCs, which might be an important effect of EMF on neuronal excitation in the cerebellar GCs. Thus, a thorough investigation of the influence of ELF-EMF on Na_v_ channels and the corresponding mechanism of action could elucidate the ELF-EMF-induced biological effects on brain physiology, pathogenesis and neural development. Therefore, it is interesting to address whether MT can modulate ELF-EMF-induced Na_v_ channel activity.

This study was conducted to determine whether MT influences the Na^+^ channels of cerebellar GCs exposed to ELF-EMF and, if so, whether this effect is mediated by inactivation of the cAMP/PKA signalling pathway. The data presented in this report demonstrate that the activity of neuronal Na^+^ channels by ELF-EMF stimulation is significantly reversed by MT. Notably, the effect of MT on ELF-EMF-induced Na^+^ is not mediated by inhibition of the cAMP/PKA signalling pathway but by increasing intracellular calcium levels.

## Materials and methods

### Ethics statement

This study was carried out in strict accordance with the recommendations in the Guide for the Care and Use of Laboratory Animals of the National Institutes of Health. The protocol was approved by the Committee on the Ethics of Animal Experiments of Fudan University (Permit Number: 20090614-001). All surgery was performed under sodium pentobarbital anaesthesia, and all efforts were made to minimize suffering.

### Primary cell culture

Cells were derived from the cerebellum of 7-day-old Sprague–Dawley rat pups as described previously [26]. Isolated cells were then plated onto 35-mm-diameter Petri dishes coated with poly-l-lysine (1 μg/ml) at a density of 2.5 × 10^5^/cm^2^. Cultured cells were incubated at 37°C with 5% CO_2_ in DMEM supplemented with 10% foetal calf serum, glutamine (5 mM), insulin (5 μg/ml), KCl (25 mM) and 1% antibiotic–antimycotic solution. All experiments were carried out with cerebellar GCs grown for 6–8 days in culture (DIC). For Ca^2+^ imaging experiments, the cells were plated onto poly-l-lysine-coated glass coverslips (12 mm in diameter).

### Electromagnetic field production

The system used to expose cerebellar GCs cells to electromagnetic fields was the same as that used in previous studies, with some revisions (I-ONE, Shanghai, China) [27,28]. Briefly, a 50-Hz magnetic field was generated by a pair of Helmholtz coils placed in opposition to each other. The coils were powered by a generator system that produced sinusoidal input voltage, and the magnetic flux densities could be regulated within the range of 0–1.0 mT. The device was powered by an AC power generator, and the EMF frequency and density were monitored by an EMF sensor that was connected to a digital multimeter. The geometry of the system assured a uniform field for the exposed cultured cells. The surfaces of the culture plates were parallel to the force lines of the alternating magnetic field in the solenoid. The air and culture medium temperatures were continuously monitored for the duration of experiments. The maximum temperature increase recorded in the cultures that were exposed to ELF-EMF (compared with non-exposed cultures) was 0.4 ± 0.1°C. To identify any possible influence of this increase on our results, we compared data obtained from cerebellar GCs cultured in two different CO_2_ incubators at temperature settings of 37.0 and 37.4°C, and the results were consistent. The incubator was keep closed throughout the EMF or non-EMF experiments to ensure that the conditions were stable. Non-EMF groups were incubated in the same incubator under the same conditions as those used for the exposed groups but without EMF.

### Patch-clamp recordings

Whole-cell currents of granule neurons were recorded using a conventional patch-clamp technique. In 6–8 DIC cerebellar GCs, transient *I*_Na_ are largely unclamped because of an event generated at a site electrotonically distant from the soma and prone to escape from clamp control, presumably the axon [29]. Therefore, we chose those cells that were relatively isolated and only recorded currents without unclamped spike. Prior to current recordings, the culture medium was replaced with a bath solution containing (in mM) NaCl 145, KCl 2.5, HEPES 10, MgCl_2_ 1 and glucose 10 (pH adjusted to 7.4 using NaOH). Soft glass recording pipettes were filled with an internal solution containing (in mM) CsCl 145, HEPES 10, MgCl_2_ 2 and EGTA 5 (pH adjusted to 7.3 using CsOH). The pipette resistance was 5–6 MΩ after being filled with the internal solution. Whole-cell series resistances of 6–8 MΩ were routinely compensated by more than 70%. All currents were recorded using an Axopatch 200B amplifier (Axon Instruments, Foster City, CA, USA) that was operated in voltage-clamp mode. A Pentium computer was connected to the recording equipment with a Digidata 1300 analogue-to-digital (A/D) interface. The current was digitally sampled at 100 μs (10 kHz), and the current signals were filtered using a 5-kHz, five-pole Bessel filter. The currents were corrected online for leak and residual capacitance transients using a P/4 protocol. Data acquisition and analysis were performed with pClamp10 software (Axon Instruments) and/or Origin8.1 (MicroCal, Northampton, MA, USA). All recordings were performed at room temperature (23–25C°).

### Phosphorylated protein kinase A assay

The cells were lysed in HEPES-NP40 lysis buffer (20 mM HEPES, 150 mM NaCl, 0.5% NP-40, 10% glycerol, 2 mM EDTA, 100 μM Na_3_VO_4_, 50 mM NaF and 1% proteinase inhibitor cocktail at pH 7.5) on ice for 30 min. After centrifugation, the supernatant was mixed with 2× sodium dodecyl sulphate loading buffer and boiled for 5 min. The proteins were separated on a 10% polyacrylamide gel, transferred to polyvinylidene difluoride membranes (Millipore, Billerica, MA, USA), blocked with 10% non-fat milk and incubated at 4°C overnight with a rabbit polyclonal antibody against the phosphorylated form of the PKA catalytic subunits (1:1000; Santa Cruz Biotechnology Inc., Santa Cruz, CA, USA) or a rabbit monoclonal antibody against GAPDH (1:1000; KangCheng, China). After extensive washing with TBST, the membrane was incubated with horseradish peroxidase–conjugated antimouse or anti-rabbit IgG (1:10,000; KangChen Bio-Tech, Shanghai, China) for 2 hrs at room temperature. Chemiluminescent signals were generated using a SuperSignal West Pico trial kit (Pierce, Rockford, IL, USA) and detected using a ChemiDoc XRS System (Bio-Rad Laboratories Inc., Hercules, CA, USA). The protein measurements were normalized with GAPDH and control/GAPDH as 1.0.

### Measurement of intracellular Ca^2+^ levels

Intracellular Ca^2+^ levels were detected by single cell fura-2 AM fluorescence intensity as described by Grynkiewicz [30]. Briefly, cultured cerebellar GCs were rinsed twice with balanced salt solution (BSS), then incubated at 37°C for 45 min. in the presence of 5 μM fura-2 AM (0.1% dimethylsulfoxide (DMSO) in BSS), washed twice again with BSS and incubated for an additional 20 min. prior to imaging. The BSS was composed of (in mM) NaCl 145, KCl 2.5, HEPES 10, MgCl_2_ 1, glucose 10, CaCl_2_ 2. The coverslips were transferred to a coverslip chamber, which was mounted on the stage of an inverted phase contrast microscope (Nikon, Eclipse Ti, Japan). Fresh BSS was added to the chamber, and the data were collected at 4-sec. intervals throughout the experiment. The excitation wavelengths for fura-2 AM were 340 and 380 nm, with emission at 505 nm. Baseline [Ca^2+^]_i_ was determined for 60 sec. immediately prior to the addition of high K^+^ solution (27 mM KCl). Quantification of the fluorescence intensity was performed with Metafluor software (Universal Imaging Corporation, Downingtown, PA, USA).

### Statistical analysis

Statistical analysis was performed with Student's *t*-test with non-paired or paired comparisons, as relevant. The values are given as the means ± SEM, with *n* representing the number of cells tested. A value of *P* < 0.05 was considered a significant difference between groups. When multiple comparisons were made, the data were analysed by one-way anova followed by the Tukey and Fisher LSD test for samples of more than two using Originpro software (OriginLab Corporation, Northampton, MA, USA).

## Results

**Figure 1:**
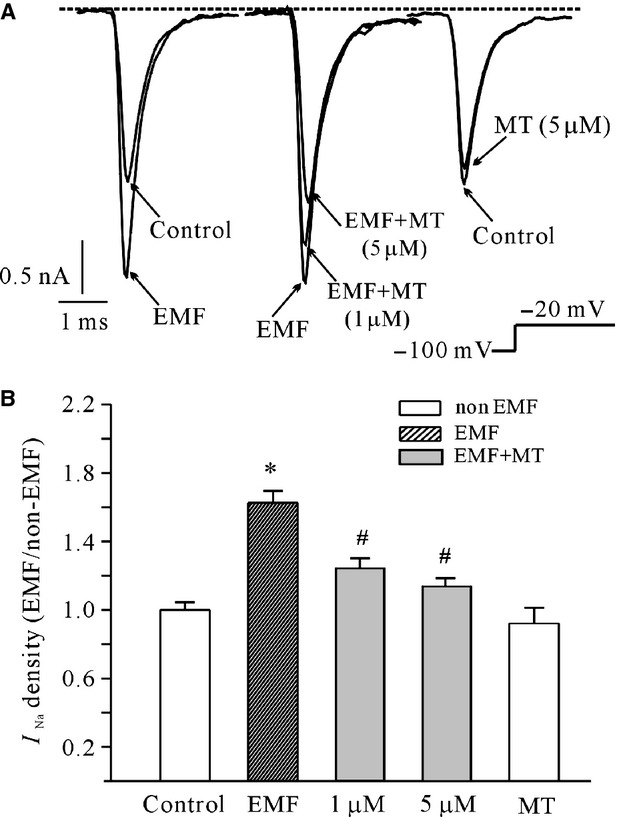
The effects of melatonin (MT) on increased *I*_Na_ densities induced by exposure to extremely low-frequency electromagnetic field (ELF-EMF). (A) Superimposed *I*_Na_ evoked by a 20-msec. depolarizing pulse from a holding potential 100 to −20 mV. Current traces were obtained from cerebellar granule cells exposed to 1 mT ELF-EMF for 60 min. in the presence or absence of MT (1 and 5 μM). (B) Statistical analysis of the effects of MT on increased *I*_Na_ densities induced by exposure to ELF-EMF. The data are reported as the means ± SEM from 16 to 25 cells. *P < 0.05 compared with control (non-ELF-EMF) using Student's *t*-test. #*P* < 0.05 compared with the corresponding ELF-EMF control (without MT) using Student's *t*-test.

First, we investigated the effect of MT on the influence of ELF-EMF on the *I*_Na_ of cerebellar GCs. An *I*_Na_ was elicited by a depolarization step to −20 mV from the holding potential of −100 mV. Our previous study demonstrated that the increase in *I*_Na_ amplitude induced by ELF-EMF exposure was time dependent, and when cerebellar GCs were exposed to 1 mT ELF-EMF for 60 min., the amplitude of the *I*_Na_ increased significantly and was stable [10]. Moreover, exposure cell or neuron to 1–5 mT EMF with short time was reported by Bai and Moghadam's studies [31,32]. Therefore, we chose the same parameters of 1 mT ELF-EMF for 60 min. in this study. Similar to our previous report [10], when cerebellar GCs were exposed to 1 mT ELF-EMF for 60 min., the amplitude of the *I*_Na_ increased by ∽62.5 ± 6.6% (*n* = 25, *P* < 0.05) compared with cells that were not exposed to ELF-EMF (*n* = 33, Fig.1A). Melatonin significantly inhibited the increase in *I*_Na_ induced by ELF-EMF. In the presence of 1 μM or 5 μM MT, 60 min. of ELF-EMF exposure only increased the *I*_Na_ by 22.0 ± 5.9% and 8.9 ± 4.4% (*n* = 15 and 16, *P* < 0.05), respectively, which is significantly different from 60 min. of ELF-EMF exposure alone. However, MT alone did not modify *I*_Na_ activity. There was no significant difference from the control group when 5 μM MT was added to the bath solution (Fig.1B).

**Figure 2:**
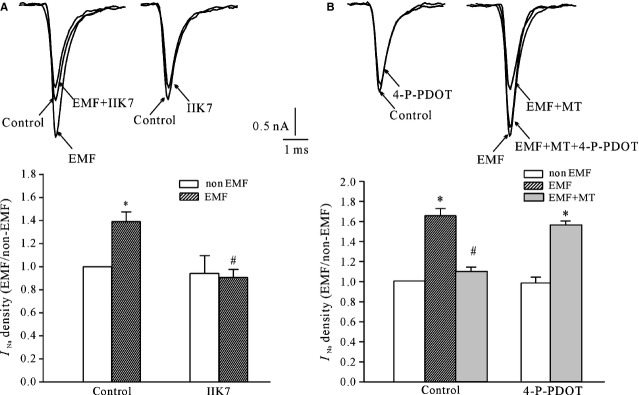
The effects of MT_2_ receptor agonist and antagonist on the melatonin (MT)-mediated inhibitory effects on extremely low-frequency electromagnetic field (ELF-EMF) exposure–induced *I*_Na_ enhancement. (A) Current traces and statistical analysis show the effects of the selective MT_2_R agonist IIK7 on *I*_Na_ obtained from ELF-EMF- and non-ELF-EMF-exposed groups. (B) Current traces and statistical analysis show the effects of the selective MT_2_R antagonist 4-P-PDOT on MT-induced inhibition of *I*_Na_ in ELF-EMF-exposed cerebellar granule cells. *P < 0.05 compared with the corresponding control (non-ELF-EMF exposed) using Student's *t*-test. #*P* < 0.05 compared with ELF-EMF exposed without MT using Student's *t*-test.

The inhibitory effect of MT on the ELF-EMF-induced *I*_Na_ activity could be mimicked by the MT_2_ receptor agonist, IIK-7 (Fig.2A). Similar to MT, IIK7 alone did not affect *I*_Na_ activity. In the presence of 10 μM IIK7, the increase in *I*_Na_ induced by ELF-EMF exposure was reduced from 46.8 ± 3.5% (*n* = 20) to −9.2 ± 6.5% (*n* = 27). This indicates that when IIK7 was used, the *I*_Na_ densities obtained from exposed cerebellar GCs were reduced by 9.2 ± 6.5% compared with that of non-ELF-EMF exposure group. Blocking MT_2_ with 4-P-PDOT eliminated the inhibitory effect of MT on ELF-EMF-induced *I*_Na_ activity. Pre-incubation of cerebellar GCs with 4-P-PDOT (10 μM) in the medium resulted in an increase in the *I*_Na_ amplitude after exposure to ELF-EMF by 56.3 ± 3.0% (*n* = 24). These data were significantly different from that obtained from cells exposed to ELF-EMF with MT (*n* = 25, Fig.2A). However, 4-P-PDOT (10 μM) itself did not modify the *I*_Na_ amplitude (Fig.2B).

**Figure 3:**
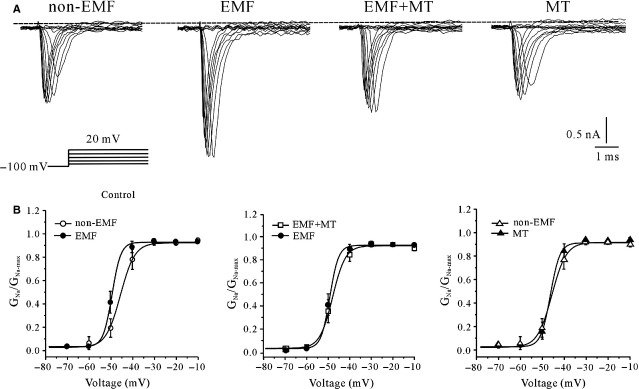
The effect of melatonin (MT) on the steady-state activation property of *I*_Na_ channels in extremely low-frequency electromagnetic field (ELF-EMF)-exposed and non-ELF-EMF-exposed cerebellar granule cells (GCs). (A) Representative superimposed *I*_Na_ evoked by steady-state activation voltage protocol obtained from non-ELF-EMF- and ELF-EMF-exposed cerebellar GCs in the presence and absence of MT. The cells were held at −100 mV and depolarized in 5-mV steps from −70 to 20 mV with intervals of 5 sec. (B) The voltage-dependent activation curve of *I*_Na_ obtained from non-ELF-EMF- and ELF-EMF-exposed cerebellar GCs in the presence and absence of MT. The data are expressed as the means ± SEM from 13 to 12 cells.

Our previous data indicated that ELF-EMF exposure significantly shifted the voltage dependence of the steady-state activation curve of *I*_Na_, but the steady-state inactivation curve of *I*_Na_ did not significantly shift upon exposure to ELF-EMF [10]. We further investigated whether the inhibitory effects of MT on ELF-EMF-induced *I*_Na_ activity were because of modulation of the voltage-gating properties of *I*_Na_ channels. The activation properties of *I*_Na_ in cerebellar GCs following exposure to ELF-EMF were studied using the appropriate voltage protocols. *I*_Na_ was evoked by a 20-msec. depolarizing pulse from a holding potential of −100 mV to potentials between −70 and 20 mV, with 5-mV steps in 5-sec. intervals (Fig.3A). A value for the steady-state activation of *I*_Na_ was then obtained by normalizing the conductance as a function of the command potential; conductance was calculated as *G*_Na_ = *I*_Na_/(V_m1/2_ − V_rev_). The data points were fitted to the Boltzmann function *G*_Na_/*G*_Na-max_ = 1/{1+exp [(V_m1/2_ − V_m_)/k]}, and the half-activation potentials were calculated. Figure3B illustrates the steady-state activation curve of *I*_Na_ obtained from cerebellar GCs that were exposed to ELF-EMF with or without MT. The half-activation potentials obtained from the control GC group was −43.3 ± 2.1 mV, which shifted to −48.8 ± 1.3 mV (*n* = 14, *P* < 0.05) when cerebellar GCs were exposed to ELF-EMF. In the presence of MT, the half-activation potentials were −44.2 ± 1.1 mV for GCs with no ELF-EMF exposure (*n* = 6) and −47.7 ± 1.7 mV for GCs exposed to ELF-EMF (*n* = 12). These data suggest that MT did not modify the steady-state activation property of the *I*_Na_ channels of the cerebellar GCs regardless of whether they were exposed to ELF-EMF.

**Figure 4:**
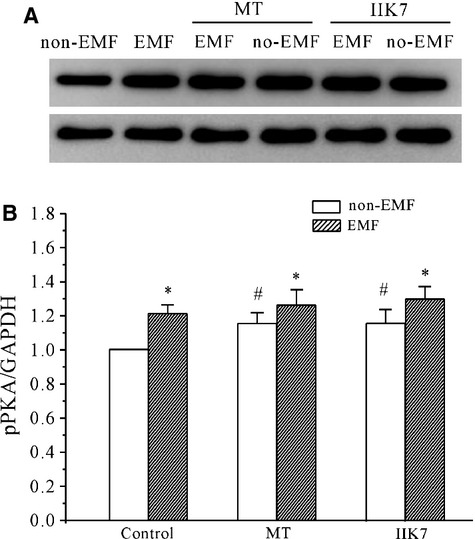
The effect of melatonin (MT) and IIK-7 on the PKA activity in extremely low-frequency electromagnetic field (ELF-EMF)- and non-ELF-EMF-exposed cerebellar granule cells (GCs) measured by Western blot analysis. (A) Representative samples show the effects of MT and IIK-7 on PKA levels. (B) The statistical analysis of the effects of MT and IIK-7 on PKA levels in ELF-EMF- and non-ELF-EMF-exposed cerebellar GCs. *P < 0.05 compared with non-ELF-EMF-exposed controls using Student's *t*-test. #*P* < 0.05 compared with the corresponding non-ELF-EMF-exposed control without MT or IIK-7 using Student's *t*-test.

Our previous study showed that the *I*_Na_ of cerebellar GCs was enhanced by activation of PKA [33], and a significant increase in the intracellular levels of phosphorylated PKA (pPKA), as measured using an immunoblot assay, was observed following ELF-EMF exposure [10]. We thus studied the effect of MT on intracellular pPKA levels to address whether MT functioned by inhibiting the PKA activity. Unexpectedly, both MT and IIK-7 increased the intracellular pPKA by 15.1 ± 5.0% and 16.2 ± 7.3% respectively (*n* = 8, *P* < 0.05; Fig.4A and B). The presence of MT or IIK-7 did not inhibit the ELF-EMF exposure-induced increase in intracellular pPKA (Fig.4A and B), which was 26.3 ± 8.7% for MT and 29.4 ± 7.3% for IIK-7 (*n* = 8, *P* < 0.05). These results suggest that PKA activation is not associated with the inhibitory effect of MT on the ELF-EMF exposure–induced increase in *I*_Na_ channel activity.

**Figure 5:**
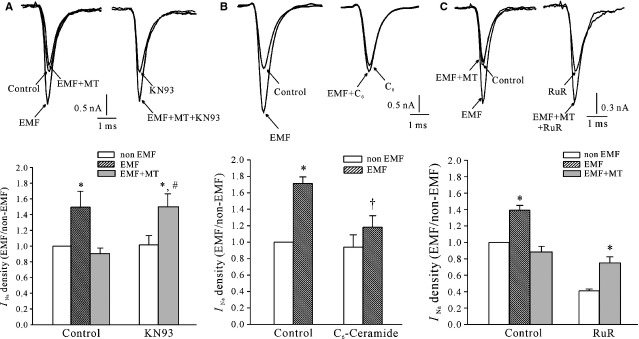
Current traces and statistical analysis show the effects of the Ca^2+^/CaM pathway on melatonin (MT)-induced inhibition of *I*_Na_ in extremely low-frequency electromagnetic field (ELF-EMF)-exposed cerebellar granule cells. (A) Representative superimposed *I*_Na_ traces and statistical analysis showing that KN-93 significantly abolished the effect of MT on the EMF-induced *I*_Na_ increase. (B) Representative superimposed *I*_Na_ traces and statistical analysis showed that C_6_-ceremade (C_6_) could mimic the effect of MT on the EMF-induced *I*_Na_ increase. (C) Representative superimposed *I*_Na_ traces and statistical analysis showed that ruthenium red (Rud) significantly abolished the effect of MT on the EMF-induced *I*_Na_ increase. *P < 0.05 compared with the non-ELF-EMF-exposed controls using Student's *t*-test. #*P* < 0.05 compared with the corresponding control (ELF-EMF exposed to MT) using Student's *t*-test. †*P* < 0.05 compared with the corresponding control without C_6_-ceramide treatment using Student's *t*-test.

It has previously been reported that Ca^2+^/CaM can modulate voltage-gated Na^+^ channels in neurons and muscles [34,35]. We therefore examined the effects of KN-93, a Ca^2+^/CaMKII blocker, on the inhibitory effect of MT on the ELF-EMF exposure–induced increase in *I*_Na_ channel activity. KN-93 alone did not modify the *I*_Na_ amplitude. In the presence of KN-93 (10 μM), the *I*_Na_ amplitude after ELF-EMF exposure with MT was increased from 8.9 ± 4.4% to 49.4 ± 17.1% (*n* = 19; Fig.5A), which was significantly different from the results obtained without KN-93 (*P* < 0.05), suggesting that the Ca^2+^/CaM pathway is associated with the inhibitory effect of MT on the ELF-EMF exposure–induced increase in *I*_Na_. We then tested the effect on ELF-EMF exposure–induced increase in *I*_Na_ by treatment of C_6_-ceramide, which increases Ca^2+^ release through the ryanodine-sensitive Ca^2+^ receptor [35]. Based on our results, C_6_-ceramide could mimic the effect of MT and decreased the ELF-EMF-induced inhibitory effect in *I*_Na_ from 71.2 ± 8.0% to 16.8 ± 11.7% (*n* = 11, Fig.5B). In rat cerebellar GCs, intracellular Ca^2+^ is mainly released by the ryanodine-sensitive Ca^2+^ receptor pathway [35]. Therefore, we used ruthenium red, a ryanodine-sensitive receptor blocker, to probe whether the inhibitory effect of MT on the ELF-EMF exposure–induced increase in *I*_Na_ is through ryanodine-sensitive Ca^2+^ receptor–induced calcium release. Consistent with previous reports by Claire O. Malecot *et al*. [36], incubation of rat cerebellar GCs with ruthenium red (10 μM) alone significantly reduced *I*_Na_ amplitude. On the other hand, blocking ryanodine-sensitive Ca^2+^ receptor by ruthenium red significantly eliminated the inhibitory effect of MT on the ELF-EMF exposure–induced increase in *I*_Na_: in the presence of ruthenium red (10 μM), the *I*_Na_ amplitude after ELF-EMF exposure with MT was still increased 85.4 ± 8.1% (*n* = 9) compared with the group treated with ruthenium red alone (Fig.5C).

**Figure 6:**
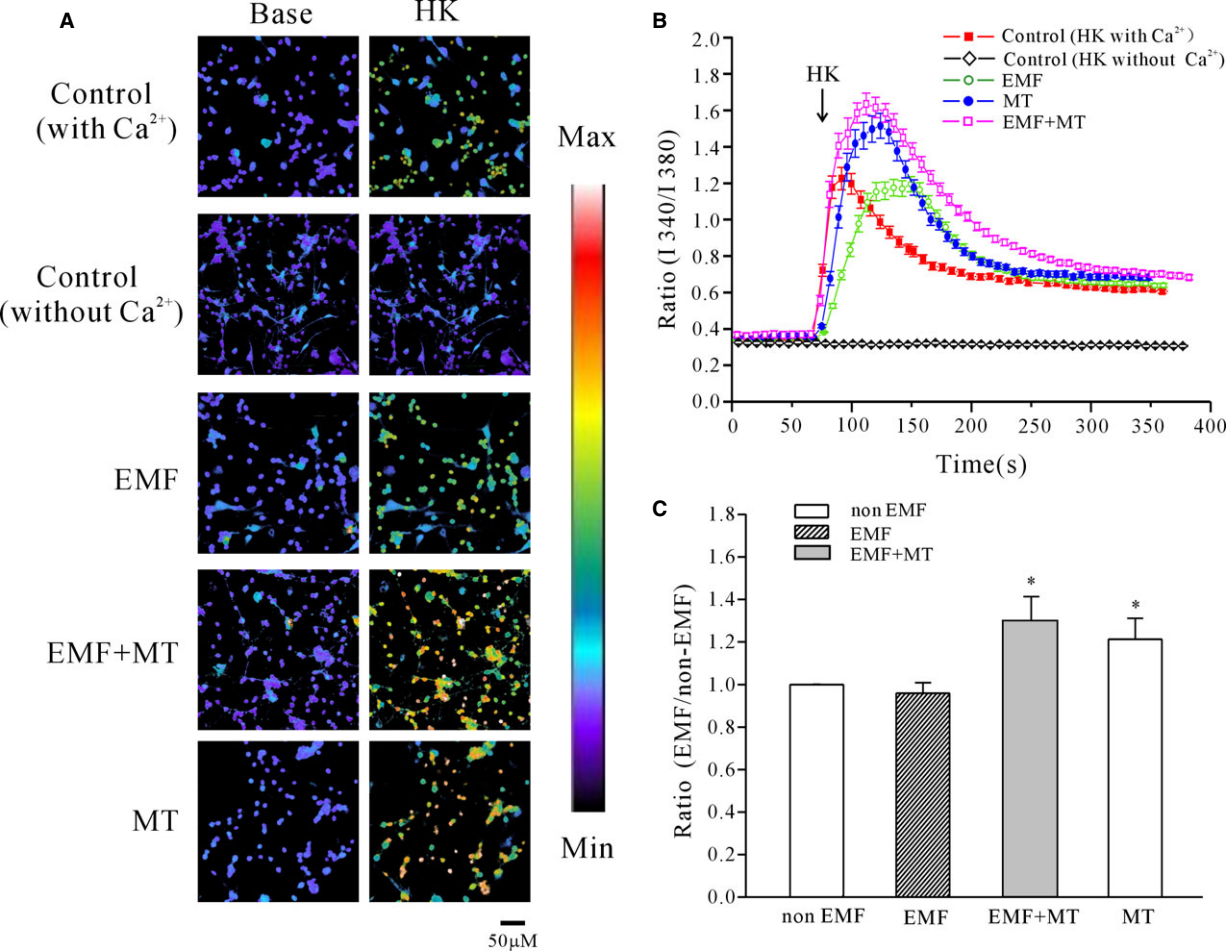
Effect of melatonin (MT) on the increase in intracellular Ca^2+^ level induced by high K^+^ in control cells and cells exposed to extremely low-frequency electromagnetic field (ELF-EMF). (A) [Ca^2+^] imaging obtained before and after depolarizing membranes by acute perfusion of a solution containing 27 mM K^+^ from ELF-EMF exposure and control cerebellar granule cells (GCs) in the presence or absence of MT. Changes in the fura-2 AM fluorescence excitation ratios with increasing [Ca^2+^] are depicted as a switch from purple to red; scale bar, 50 μm. (B) Changes in intracellular Ca^2+^ concentrations upon application of a depolarizing stimulus as measured by quantification of fluorescence excitation ratios. Each arrow represents a 30-sec. perfusion with a depolarizing solution containing 27 mM K^+^. (C) Statistical analysis of intracellular Ca^2+^ level obtained fromELF-EMF-exposed and control cerebellar GCs in the presence or absence of MT. The data were obtained from four independent experiments and are the means ± SEM; *P < 0.05 compared with the corresponding control by unpaired *t*-test.

To confirm that intracellular Ca^2+^ was associated with the inhibitory effect of MT on ELF-EMF-induced *I*_Na_ activity, we used direct Ca^2+^ imaging using the calcium-sensitive fluorescent dye fura-2 AM to examine the potential effects of MT on intracellular calcium levels in cerebellar GCs. Because MT did not affect the basal level of intracellular calcium of cerebellar GCs, we used a high K^+^-solution (27 mM KCl) to depolarize neurons and activate voltage-dependent Ca^2+^ channels (VGCCs), leading to a rapid increase in the calcium concentration [37]. In control neurons, depolarization stimulation by high K^+^ evoked an acute elevation of intracellular Ca^2+^ levels with an increase in the F340/F380 ratio from 0.37 ± 0.006 to 1.25 ± 0.061 (*n* = 26), depicting as a shift from purple to red. After exposure to ELF-EMF, the high K^+^ stimulation evoked a similar intracellular Ca^2+^ level increase from 0.35 ± 0.016 to 1.21 ± 0.055 (*n* = 25), which was not significantly different compared with the group with non-ELF-EMF exposure (Fig.6A and B). Melatonin did not increase the basal intracellular Ca^2+^ level, but it significantly elevated the intracellular Ca^2+^ level evoked by high K^+^ stimulation in cerebellar GCs that were either exposed to ELF-EMF or not (Fig.6A and B). In the presence of MT, the intracellular Ca^2+^ F340/F380 ratio evoked by high K^+^ stimulation was significantly increased in cerebellar GCs that were either exposed to ELF-EMF or not to 1.62 ± 0.064 (*n* = 35) and 1.52 ± 0.055 (*n* = 31), respectively (Fig.6B), which was significantly different from the 1.21 ± 0.055 and 1.25 ± 0.061 recorded in the absence of MT. Increases in intracellular Ca^2+^ levels were calculated as a percentage of the control based on the F340/F380 ratio and were 29.6 ± 11.0% and 21.2 ± 9.6%, respectively, (Fig.6C) for cerebellar GCs that were either exposed to ELF-EMF or not. However, when Ca^2+^ was removed from bath solution, depolarization stimulation by high K^+^ did not increase the intracellular Ca^2+^ levels, suggesting that extracellular Ca^2+^ influx was needed for the depolarization-induced intracellular Ca^2+^ increase (Fig.6A–C).

**Figure 7:**
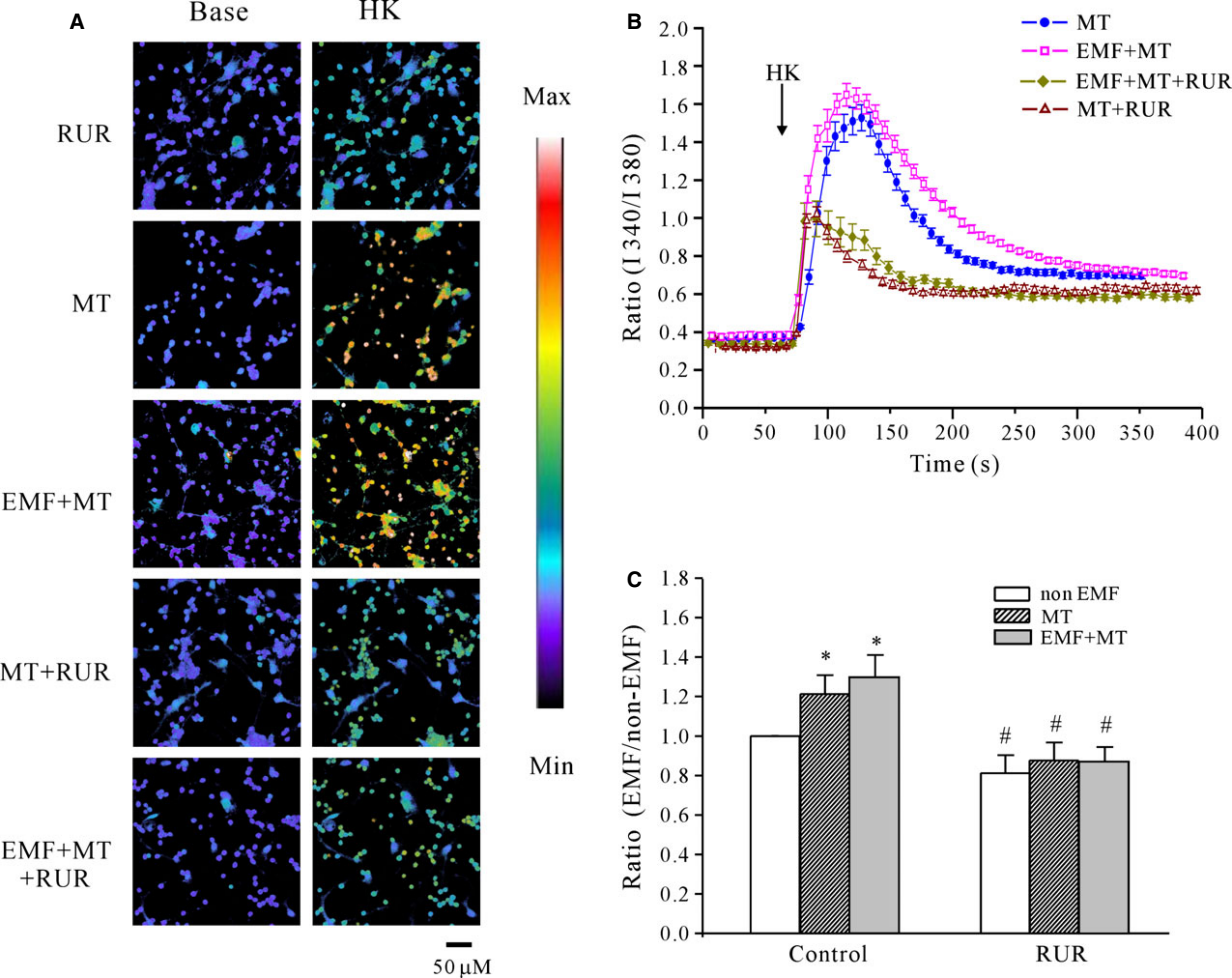
Effects of blocking ryanodine-sensitive receptors on melatonin (MT)-induced increase in intracellular Ca^2+^ level in control cells and cells exposed to extremely low-frequency electromagnetic field (ELF-EMF). (A) [Ca^2+^] imaging obtained before and after depolarizing membranes by acute perfusion of a solution containing 27 mM K^+^ from ELF-EMF-exposed cells with MT in the presence or absence of Ruthenium Red (RuR, 20 μM); scale bar, 50 μm. (B) Changes in intracellular calcium concentrations upon application of RuR. Each arrow represents a 30-sec. perfusion with a depolarizing solution containing 27 mM K^+^. (C) Statistical analysis of intracellular Ca^2+^ levels obtained after ELF-EMF exposure with MT in the presence or absence of RuR. The data are the means ± SEM obtained from four independent experiments; *P < 0.05 compared with non-ELF-EMF-exposed cells without MT using Student's *t*-test. #*P* < 0.05 compared with the corresponding control by unpaired *t*-test.

We also used ruthenium red to address whether the MT-induced increase in calcium release occurred through the ryanodine-sensitive Ca^2+^ receptor. Pre-incubation of cerebellar GCs with 20 μM ruthenium red alone induced a slight reduction in the F340/F380 ratio stimulated by high K^+^ from 1.25 ± 0.061 to 0.91 ± 0.050 (*n* = 28; Fig.7A and B), which was an inhibition of 19.2 ± 8.8% compared with the control group (Fig.7C); in the presence of ruthenium red, the increase in the F340/F380 ratio induced by MT was reduced from 1.52 ± 0.055 to 1.01 ± 0.037 (*n* = 24) in the control group. Similarly, ruthenium red also significantly inhibited the effect of MT on intracellular Ca^2+^ release after ELF-EMF exposure, which resulted in a decrease in the F340/F380 ratio from 1.62 ± 0.064 to 1.07 ± 0.078 (*n* = 34; Fig.7C).

## Discussion

Although several ion channels, such as delayed rectifier outward K^+^ current (*I*_K_), TRPM-2 and voltage-dependent L- type Ca^2+^ channels, are known to be modulated by MT [18–21], the effects of MT on Na_v_ channels are poorly understood. Although MT has previously been reported to protect cells against EMF stimulus, whether it modulates the activity of ion channels induced by EMF exposure is poorly understood. Here, we report for the first time that MT itself was not able to modify *I*_Na_, but might inhibit *I*_Na_ enhancement resulting from ELF-EMF exposure in cerebellar GCs by increasing the concentration of intracellular Ca^2+^.

It is well known that the effects of exposure to EMF differ significantly based on the ELF-EMF exposure intensities and the exposure time. Our previous study indicates that exposure of cerebellar GCs to ELF-EMF (1 mT) for short time (10–60 min.) significantly increases the amplitude of the *I*_Na_. Moreover, exposure to ELF-EMF induces similar effects on *I*_Na_ in rat cerebellar GCs regardless the condition, whether it is 1 mT stimulation for a short time or 0.4 mT stimulation for a longer time. Notably, it is generally believed that short-term changes induced by EMF are mediated by modifications in enzyme activity in the cytosol or the membrane [4,38,39], while the long-term exposure to EMF may induce changes in nuclear functions such as gene transcription and cell cycle regulation [27,40]. To avoid the influence of multiple factors because of long-term EMF exposure, we performed all our experiments at 1 mT EMF exposure for a short time, which we believe that it is suitable to assess the effect of ELF-EMF on intracellular signalling pathways.

Although some of the main functions attributed to MT include its role as a free radical scavenger and its indirect antioxidant properties [41], studies have shown that MT can interact with specific receptors to exert its biological effect [42]. Our previous study demonstrated that activation of the MT_2_ receptor (MT_2_R) by MT and a low concentration of 2-iodomelatonin increased the delayed-rectified outward K^+^ current (*I*_K_) [43] and improved cerebellar GC migration [21]. In contrast, a high concentration of 2-iodomelatonin could inhibit the *I*_K_ recorded from cerebellar GCs by activating the MT_1_ receptor, which protects the neurons against apoptotic stimulus [20]. In this study, a selective MT_2_ agonist could mimic the effect of MT on EMF-induced *I*_Na_ enhancement, which could be blocked by a selective MT_2_ antagonist, suggesting that it is highly likely that the inhibitory effect of MT on EMF-induced *I*_Na_ enhancement was mediated by MT_2_R, and did not directly result from the antioxidant properties of MT. This is consistent with our previous findings on the effect of MT on the potassium current in cerebellar GCs [20,21,43].

Among the possible mechanisms underlying the inhibitory effect of MT on EMF-induced *I*_Na_ increase, we first considered the involvement of the MT_2_-mediated cAMP/PKA pathway because EMF-induced *I*_Na_ increase is thought to occur *via* the cAMP/PKA pathway [10], and negative modulation of cAMP/PKA activation by MT has previously been reported [21]. However, the experimental data presented here show that MT did not inhibit PKA activity; instead, it induced a slight increase in PKA activity. Furthermore, MT was not able to inhibit the PKA activity induced by EMF exposure. In addition, MT did not change the steady-state activity of *I*_Na_ channels in the control cerebellar GCs or in EMF-exposed cerebellar GCs, indicating that a non-phosphorylation-dependent mechanism is involved. Together, these data suggest that the ability of MT to counteract the effect of EMF on *I*_Na_ activity does not occur through the inhibition of EMF-induced PKA activity. MT failed to negatively modulate PKA activity in this study, which is different from what has previously been reported [21]. This effect might be as a result of the higher concentration of MT used in this study. Transfection experiments have demonstrated that when expressed in the same cell type, MT_1_ and MT_2_ receptors may couple to different signalling pathways [44]. Both our work and that of Marta indicate that the presence of native MT_1_/MT_2_ receptors in mouse and rat cerebellar GCs mediates the effects of MT on intracellular signalling pathways [20,21,45]. Thus, it is possible that 1–5 μM MT may activate both MT_1_ and MT_2_ receptors simultaneously, thereby inducing a coordinated and integrated effect on PKA. Therefore, the effect of MT on the PKA pathway was no longer evident.

In addition to the cAMP/PKA pathway, Ca^2+^ has been proposed to regulate Na^+^ channels through the action of calmodulin (CaM) bound to an isoleucine–glutamine motif in the C terminus of the Na^+^ channel subunit [46]. Previous studies indicate that MT modulates the Ca^2+^/CaM signalling pathway either by changing the intracellular calcium concentration ([Ca^2+^]_i_) *via* activation of its G-protein coupled membrane receptors or through a direct interaction with CaM [47,48]. MT was shown to be able to reverse cytosolic Ca^2+^ evoked by H_2_O_2_ stimuli [19]. In rat cerebellar GCs, intracellular Ca^2+^ is mainly released by the ryanodine-sensitive Ca^2+^ receptor pathway, which might maintain *I*_Na_ at a lower level [34]. In this study, the Ca^2+^/CaMKII and ryanodine-sensitive Ca^2+^ receptor blocker significantly abolished the effect of MT on the EMF-induced *I*_Na_ increase, providing evidence for the involvement of the Ca^2+^/CaM pathway. Interestingly, MT did not increase basal Ca^2+^ release, but significantly increased high K^+^ evoked intracellular Ca^2+^ levels, which was thought to result from membrane depolarization [49]. Furthermore, the increase in intracellular Ca^2+^ evoked by MT was inhibited when the ryanodine-sensitive Ca^2+^ receptor was blocked with ruthenium red. It is thus highly likely that MT activated the MT_2_R-mediated Ca^2+^ channels and improved extracellular Ca^2+^ influx, which consequently stimulated the release of Ca^2+^ through the ryanodine-sensitive Ca^2+^ receptor, by which the EMF-induced *I*_Na_ increase was reversed.

However, our previous study with Western blotting has revealed [10] that ELF-EML increases the current density by increasing the Na_V_ channel protein expression on cerebellar GCs membrane, although the content of expression is lower. Whether increase in Na_v_ channel protein expression or else mechanism is involved in MT-induced and Ca^2+^/CaM-dependent modulation of *I*_Na_ amplitude remains unclear, and further study is necessary.

Taken together, our data suggest that MT eliminated the EMF-induced *I*_Na_ increase through Ca^2+^ influx–induced Ca^2+^ release but not by abolishing EMF-induced PKA activity. However, we noticed that MT itself did not affect the amplitude of *I*_Na_ in control cerebellar GCs, although it was able to increase the intracellular Ca^2+^ levels evoked by high K^+^ depolarization stimuli. This might be because basal intracellular Ca^2+^ released from ryanodine-sensitive receptors maintain the *I*_Na_ at a low enough level in rat cerebellar GCs [34] that MT-induced Ca^2+^ release is not able to reduce the *I*_Na_ densities further. However, when cerebellar GCs were exposed to EMF with MT, high K^+^-mediated Ca^2+^ release was significantly enhanced, and MT reversed the EMF-induced increase in *I*_Na_ amplitude. This observation was consistent with previous studies that indicated that most of the effects of MT on second messengers or effectors require prior stimulatory input [50,51]. It is highly likely that with EMF exposure as stimulatory input, the effect of MT on *I*_Na_ could be fully realized.

Currently, there is widespread use of EMR-emitting devices in industrial, scientific, medical and domestic applications, with the potential for leakage of such radiation into the environment [52]. The effects of ELF-EMF on nerve cells have been extensively studied in various organisms [8,53]. Although the reported results are variable or contradictory because of differences in the experimental conditions and in the density and/or duration of EMF exposure, EMF has recently been reported to modulate neuronal excitatory functions and neurogenesis [8,24,25]. The findings from our molecular-level analysis of the protective effects of MT on *I*_Na_ produced by exposure to ELF-EMF might provide evidence for an important effect of MT on neuronal excitation during EMF exposure in cerebellar GCs. Nonetheless, the modulatory effects on neuronal excitatory functions caused by exposure to ELF-EMF are complicated and varied. In addition, the functional mechanism of MT and its receptors in native cerebellar GCs are complex. Therefore, further exploration is required to comprehensively analyse the biological protective effects of MT and its mechanism on neurons that are exposed to ELF-EMF.
